# The effect of N-acetylcysteine in patients with non-cystic fibrosis bronchiectasis (NINCFB): study protocol for a multicentre, double-blind, randomised, placebo-controlled trial

**DOI:** 10.1186/s12890-022-02202-9

**Published:** 2022-11-07

**Authors:** Yue Liao, Yanqiu Wu, Kai Zi, Yongchun Shen, Tao Wang, Jiangyue Qin, Lei Chen, Mei Chen, Lin Liu, Weiming Li, Hui Zhou, Shuguan Xiong, Fuqiang Wen, Jun Chen

**Affiliations:** 1 Division of Pulmonary Disease, Department of Respiratory and Critical Care Medicine, West China Hospital of Sichuan University, Chengdu, China; 2 Department of Respiratory, Chengdu Fifth People’s Hospital, Chengdu, China; 3 Department of Respiratory and Critical Care Medicine, 363 Hospital, Chengdu, China; 4 Department of Respiratory Medicine, Chengdu Sixth People’s Hospital, Chengdu, China; 5 Department of Respiratory, Chengdu University Affiliated Hospital, Chengdu, China; 6 Department of Clinical Laboratory, Nuclear Industry 416 Hospital, the 2nd Affiliated Hospital of Chengdu Medical College, Chengdu, China

**Keywords:** Non-cystic fibrosis bronchiectasis, N-acetylcysteine, Quality of life, Exacerbation, Bacterial colonization, Oxidative stress, Chronic inflammation, Clinical trial

## Abstract

**Background:**

N-acetylcysteine (NAC), which is specifically involved in airway mucus clearance and antioxidation, is recommended by the treatment guideline for non-cystic fibrosis bronchiectasis (NCFB). However, there is little clinical evidence of its long-term efficacy concerning quality of life (QoL) and exacerbation in patients with NCFB. In addition, the influences of NAC on airway bacterial colonization, chronic inflammation and oxidative stress in NCFB are also unclear.

**Methods:**

NINCFB is a prospective, multicentre, double-blind, randomised, placebo-controlled trial that will recruit 119 patients with NCFB and randomly divide them into an NAC group (*n* = 79) and a control group (*n* = 40). Participants in the NAC group will receive 600 mg oral NAC twice daily for 52 weeks, while patients in the control group will receive 600 mg placebo twice daily for 52 weeks. The information at baseline will be collected once participants are enrolled. The primary endpoints are the changes in St George’s Respiratory Questionnaire scores and the number of exacerbations in 52 weeks. The secondary endpoints are the 16S rRNA of sputum and the levels of inflammatory factors and oxidative stressors in sputum and serum. Other data related to radiography, lung function tests, number of oral and/or intravenous antibiotic therapies and adverse events (AEs) will also be analysed. Further subgroup analysis distinguished by the severity of disease, severity of lung function, airway bacterial colonization and exacerbation frequency will be performed.

**Discussion:**

The objective of this study is to determine the long-term efficacy of NAC on QoL and exacerbation of NCFB and to explore the effectiveness of NAC for antibiosis, anti-inflammation and antioxidation in NCFB. The study results will provide high-quality clinical proof for the revision and optimization of treatment guidelines and for expert consensus on NCFB treatment.

**Trial registration:**

The trial was registered on the Chinese Clinical Trial Register at April 11, 2020 (chictr.org.cn, ChiCTR2000031817).

## Background

The worldwide prevalence and health care burden of non-cystic fibrosis bronchiectasis (NCFB) have been increasing rapidly in recent years [1–3]. In China, nearly 1.2% of individuals over 40 years old are diagnosed with NCFB, and its morbidity is positively correlated with age [4]. Effective therapy and management of this disease are urgently needed.

NCFB, caused by various primary pathogenic processes, such as pathogenic organism infection, immune deficiency and congenital defects [5], is a chronic purulent lung disease causing irreversible bronchial dilation and bronchial wall thickening [6, 7], which greatly complicates its treatment and management [8]. Accumulation of sputum, chronic airway inflammation and repeated exacerbations are the main characteristics of NCFB [9, 10]. Research has shown that 50% of patients with NCFB have two or more exacerbations annually, and one in three require hospitalization [11]. Continual expectoration and frequent exacerbations have significantly troubled patients and greatly reduced their quality of life (QoL) [12]. Consequently, improving airway mucus clearance, reducing exacerbation and enhancing QoL are the significant treatment goals of NCFB [13].

N-acetylcysteine (NAC), which provides the sulfhydryl group that can break the disulfide bond of the glycoprotein polypeptide chain in the phlegm to reduce the viscosity of sputum [14, 15] and can act as a direct reactive oxygen species (ROS) scavenger to regulate the redox status in the cells [16], has been recommended by treatment guidelines of chronic pulmonary disease for airway mucus clearance [15]. However, there are only a few clinical investigations on its specific efficacy for NCFB, and the long-term effectiveness of NAC for patients’ QoL has not been reported either. In addition, although NAC was determined to have antibacterial properties [17, 18] and the ability to interfere with signal pathways of inflammation [19] and oxidative stress [20] in chronic obstructive pulmonary disease (COPD), these capabilities in NCFB are still unclear [13].

A randomised controlled trial conducted by Qi, Q., et al. in 2014 demonstrated that NAC can decrease the number of exacerbations of patients with NCFB [21], but it did not evaluate the QoL of patients or further explore the antibiosis, anti-inflammation and antioxidation of NAC for NCFB.

Given all of these findings, we designed this clinical trial (NINCFB) to test our hypothesis that NAC can play a significant role in antibiosis, anti-inflammation and antioxidation to reduce exacerbations and enhance QoL of patients with NCFB.

## Methods

### Study objective

The key objectives of NINCFB are as follows:

1. Determine the long-term efficacy of NAC in improving QoL of patients with NCFB.
2. Determine the long-term effecacy of NAC in reducing exacerbation frequency of patients with NCFB.
3. Explore the activities of NAC in antibiosis, anti-inflammation and antioxidation for the treatment of NCFB.
4. Evaluate the long-term influence of NAC on clinical manifestations, pulmonary function and computed tomographic imaging features of patients with NCFB.
5. Evaluate the long-term adverse effects of NAC on patients with NCFB
6. Conducting a subgroup analysis to investigate the different effectiveness of NAC on NCFB with different severities and causes, such as pathogen infection, immune deficiency and congenital defects.
7. Trace biomarkers that can be predictors of NCFB progression or indicators of NAC efficacy.

### Study design and intervention

**Fig. 1:**
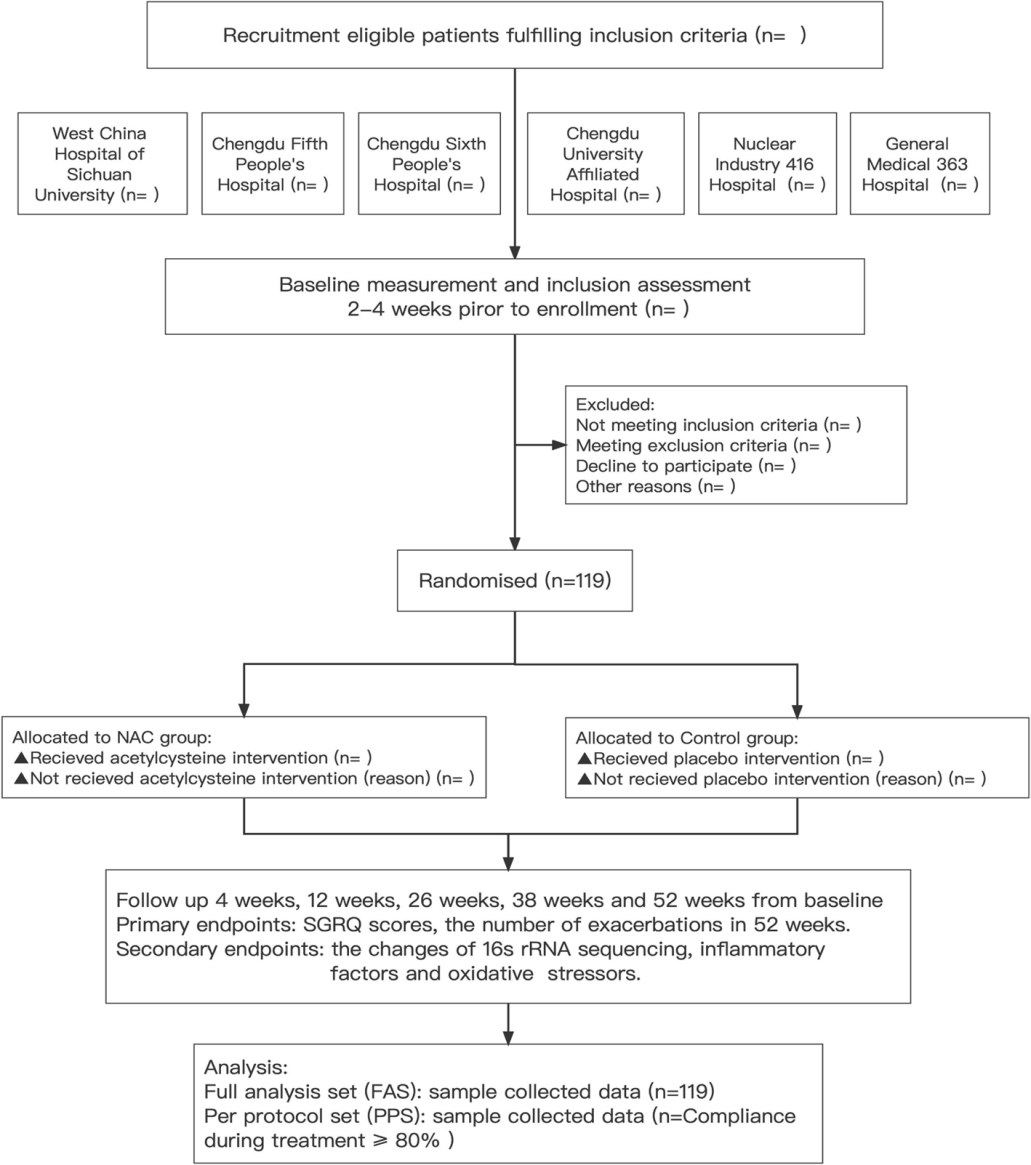
Flow chart summarising the study procedure

NINCFB is a prospective, multicentre, double-blind, randomised, parallel, placebo-controlled trial studying NCFB in Chengdu city, Sichuan Province of China (Fig. 1). It is recruiting 119 patients with NCFB from 6 hospitals located in Chendu city and randomly (2:1) assigning these patients into an N-acetylcysteine (NAC) group (*n* = 79) and a control group (*n* = 40). Participants in the NAC group will be given oral N-acetylcysteine (produced by Hainan Zambon Pharmaceutical Co., Ltd.; national medicine permission number: H20000471; 600 mg, twice daily) for 52 weeks, while the control group will receive oral placebo (produced by Hainan Zambon Pharmaceutical Co., Ltd.; 00520C0; 600 mg, twice daily) for 52 weeks. All the participants will be followed up throughout the intervention period and will be required to provide relevant information about their demography, aetiology, symptomatology, bacteriology and radiography. The results will be analysed and presented in other papers. Study enrolment started on 3 March 2021 and is expected to be completed on 31 December 2024.

### Study setting

Chengdu city is the largest city in Sichuan Province, located in southwestern China, and has a residential population of 21.2 million. We included 6 hospitals in Chengdu based on willingness to participate (Table 1). All the patients meeting the inclusion criteria from each hospital are recommended to attend and complete the trial in the Good Clinical Practice Center of West China Hospital of Sichuan University.

**Table 1:** Hospitals participating in the trial

Research Centers in Chengdu City	
West China Hospital of Sichuan University	
Chengdu Fifth People’s Hospital	
Chengdu Sixth People’s Hospital	
Chengdu University Affiliated Hospital	
Nuclear Industry 416 Hospital	
General Medical 363 Hospital	

### Study participants

Both outpatients and inpatients who satisfy the inclusion criteria and do not meet the exclusion criteria will be invited into the study. All participants are required to sign informed consent forms before trial enrolment. The original documents will be preserved by themselves, and copies will be archived with the case report forms (CRFs).

#### Inclusion criteria

(1) 18 years or older at enrolment; (2) having diagnosed with non-cystic fibrosis bronchiectasis (NCFB) by high-resolution computed tomography (HRCT) scans; (3) a forced expiratory volume in 1 s (FEV1) ≥ 30% of its predicted value in pulmonary function tests; (4) having had at least two pulmonary exacerbations requiring oral antibiotics or at least one pulmonary exacerbation necessitating intravenous antibiotics in the year before enrolment; and (5) having been in a stable state of NCFB for at least 4 weeks prior to randomisation.

#### Exclusion criteria

(1) Bronchiectasis due to cystic fibrosis; (2) having received systemic antibiotics treatment within 4 weeks before the follow-up; (3) primary diagnosis of COPD or asthma; (4) having diagnosed with allergic bronchopulmonary aspergillosis (ABPA), active nontuberculous mycobacterium (NTM) infection or active tuberculosis; (5) comorbidity with severe cardiovascular, digestive, kidney diseases and malignant tumours; (6) history of surgeries in the past 3 months prior to enrolment; (7) known allergy to NAC; (8) pregnancy or lactation or plan for pregnancy in the next 52 weeks; (9) history of prior macrolide use of more than 1 week; (10) poor compliance (any patient who has poor memories to take medicine and has no caregiver or family for supervision is considered as poor compliance.).

### Outcome measures

All outcome measures will be recorded at baseline and during the intervention period (Table 2).

**Table 2:** Study main measures and outcomes to be collected

Variable	Baseline (0-week)	1-month follow-up (4-weeks)	3-month follow-up (12-weeks)	6-month follow-up (26-weeks)	9-month follow-up (38-weeks)	12-month follow-up (52-weeks)
Primary Endpoint 1						
SGRQ scores	X	X	X	X	X	X
Primary Endpoint 2						
Number of exacerbations during the follow-up		X	X	X	X	X
Secondary Endpoints						
16S rRNA sequencing of sputum	X			X		X
Inflammatory factors	X			X		X
Oxidative stressors	X			X		X
Other Variables						
Clinical signs and symptoms	X	X	X	X	X	X
Transcutaneous regional oxygen saturation	X	X	X	X	X	X
BSI scores	X	X	X	X	X	X
mMRC dyspnea scores	X	X	X	X	X	X
HRCT grade, n (%)	X					X
Number of lesion lobes, n (%)	X					X
FEV1/FVC%	X			X		X
FEV1%	X			X		X
Number of exacerbations 12-months before the enrollment and during the trail	X	X	X	X	X	X
Time to first exacerbation		X	X	X	X	X
Time to second exacerbation		X	X	X	X	X
The longest duration of exacerbation in the last year	X					
The longest duration of exacerbation during the follow-up		X	X	X	X	X
Number of oral antibiotic therapies 12-months before the enrolment and during the trail	X	X	X	X	X	X
Number of intravenous antibiotic therapies 12-months before the enrolment and during the trail	X	X	X	X	X	X
Number of systemic glucocorticoid utilization12-months before the enrolment and during the trail	X	X	X	X	X	X
Number of hospitalization 12-months before the enrolment and during the trail	X	X	X	X	X	X

X means items should be evaluated or detected at the current follow-up

#### Primary endpoints

The primary endpoints of the NINCFB are quality of life (St George’s Respiratory Questionnaire, SGRQ scores) and the number of exacerbations during the follow-up period. The SGRQ, a comprehensive questionnaire to estimate pulmonary symptoms and living conditions, is commonly used to assess a patient’s quality of life [22, 23] and has good psychometric properties for the NCFB [24]. Exacerbation of NCFB is defined by the British Thoracic Society as acute deterioration (usually over several days) with worsening local symptoms (cough, increased sputum volume or change in viscosity, increased sputum purulence with or without increasing wheezing, breathlessness, or haemoptysis) and/or systemic upset [25]. This study has two variables designed to determine whether the long-term effectiveness of NAC achieves the treatment goal of NCFB (Table 2).

#### Secondary endpoints

The main secondary endpoint is 16S rRNA assessed in microbial testing of sputum, which is a measurement of airway bacterial colonization in NCFB. The levels of inflammatory factors, including interleukin-1-beta (IL-1β) [26], interleukin-8 (IL-8) [27], tumour necrosis factor-alpha (TNF-α) [28] and matrix metalloproteinase-9 (MMP-9) [29], and the levels of oxidative stressors, such as myeloperoxidase (MPO) [30], total antioxidant capacity (TAC) [31], superoxide dismutase (SOD) [32] and malondialdehyde (MDA) [20], in sputum and serum will also be detected through enzyme-linked immunosorbent assays (ELISAs) (Table 2).

#### Other variables

Baseline information will be collected at the time of enrolment (Table 3). Bronchiectasis severity index (BSI) scores, modified British Medical Research Council (mMRC) dyspnoea scores, HRCT grades and lung function parameters will facilitate further subgroup comparisons among patients with various pulmonary impairments. BSI scales will assess the NCFB severity by 9 items and divide it into 3 grades: low BSI score (0–4 points), moderate BSI score (5–8 points) and high BSI score (≥9 points) [33]. mMRC dyspnoea scales will be applied to patients with breathlessness [34]. The incidence of hospitalization, systemic glucocorticoid, and oral/intravenous antibiotic utilization 12-months before the enrolment and during the trial will also be monitored and recorded on CRFs (Table 2).

**Table 3:** Information at baseline and evaluation of adverse events

Variable	Baseline (0-week)	1-month follow-up (4-weeks)	3-month follow-up (12-weeks)	6-month follow-up (26-weeks)	9-month follow-up (38-weeks)	12-month follow-up (52-weeks)
Baseline						
Age	X					
Gender	X					
City of residence	X					
Educational level	X					
Work activity	X					
Body Mass Index (BMI)	X	X	X	X	X	X
Smoking status	X	X	X	X	X	X
Alcohol status	X	X	X	X	X	X
Hazardous substance exposure	X	X	X	X	X	X
Hereditary diseases	X					
Comorbidity	X	X	X	X	X	X
Concomitant medication	X	X	X	X	X	X
Adverse events						
Allergy	X	X	X	X	X	X
Electrocardiogram (ECG)	X			X		X
Gastrointestinal toxicity^a^	X	X	X	X	X	X
Renal toxicity^a^	X	X	X	X	X	X
Hematological toxicity^a^	X			X		X
Biochemical toxicity^a^	X			X		X

X means items should be evaluated or detected at the current follow-up

^a^Variables consist of clinical manifestation assessing (including nausea, vomit, abdominal distension, abdominal pain and so on) and blood tests results (containing red blood cells counts, aspartate aminotransferase/Alanine aminotransferase (AST/ALT), creatinine and others)

### Adverse events

The safety of NAC will be evaluated during the whole follow-up period. Participants will receive study information containing explicit details on whom to contact in case of adverse events (AEs). Three respiratory specialists will judge the relationship between AEs and intervention drugs and will record the dates, solutions, endings and conclusions on the CRFs. Patients with AEs will be told to discontinue the study once the causality is determined (Table 3).

### Data collection

Two clinical researchers at West China Hospital of Sichuan University will collect the data and make appointments for the following dates at the end of the first meeting to promote participant retention. A monitoring team consisting of 2 respiratory physicians and 1 investigator will verify and revise the results, it is independent from the sponsor and competing interests. Peripheral blood tests, electrocardiogram (ECG) readings, spirometry and HRCT scans for all the subjects will be conducted by laboratory experts and radiologists at West China Hospital of Sichuan University. All data collected both on paper and in electronic archives will be marked with a study identification number to confirm participants and will be stored in a locked cabinet and hospital computer system. Once recorded, the data will be locked to prevent changes. Missing data because of “no-shows” will be coded as incomplete. Access to the identified information sets will be limited to the study authors.

### Statistical analysis

The resulting data will then be analysed with SPSS V.26 by statistical experts. Primary outcomes of each group will be compared by Student’s t test (t test) or the Wilcoxon rank-sum test after calculating its normal distribution and will be expressed in terms of the mean, standard deviation (SD) or median to indicate the long-term efficacy of NAC for NCFB. The comparison between the NAC group and the control group in terms of 16S rRNA and the levels of inflammatory factors and oxidative stressors will be of vital importance. In addition, NINCFB will also use the t-test, chi-square test and Wilcoxon rank-sum test to evaluate baseline discrepancy and other variables. Regression analysis and the correlation coefficient may confirm the predictors of disease progression and indicators of NAC efficacy.

### Subgroup analysis

This trial will perform subgroup analysis by differentiating the participants according to 1) BSI scores (mild: 0–4 points, moderate: 5–8 points, severe: ≥ 9 points); 2), airway bacterial colonization (*Pseudomonas aeruginosa*, *Staphylococcus aureus*, and others); 3), number of lesion lung lobes (lesion lung lobes < 3 or ≥ 3); 4) FEV_1_/FVC (FEV_1_/FVC ≥ 70% or < 70%); 5) number of exacerbations in the 12 months prior to the start of the study (exacerbations frequency: ≤ 2 times per year, and > 2 times per year).

### Sample size

We calculate the sample size required for the evaluation of primary outcome-1 (SGRQ scores) and primary outcome-2 (number of exacerbations) respectively. As NAC is effective in reducing sputum of patients with NCFB in clinical practice, we design a ratio of 2:1 for the sample size of intervention group to control group in this study so that two-thirds of enrolled patients could receive effective treatments. For the SGRQ score, we regard mean difference (MD) of 6 points of SGRQ score as the smallest clinically important difference between two group with a standard deviation (SD) of 8.5 used in the calculation, according to the previous report of NAC on patients with COPD [35–37]. Based on 90% power with a two-sided significance level of 5%, a sample size of 64 for intervention group and 32 for control group is needed as calculated by the software PASS 15. In addition, assuming 20% of patients in each group may drop out or lose data integrity, we need to recruit 79 in the intervention group and 40 in the control group. For the number of exacerbation, we regard MD of 0.7 times of exacerbation per year as the smallest clinically important difference between two group with a SD of 1 used in calculation, according to the published study of NAC on patients with postinfection or idiopathic bronchiectasis [21]. Considering 20% dropout, we also need to recruit 79 patients in NAC group and 40 patients in placebo group (α = 0.05, 1 - β = 0.9). Taken together, this study eventually aims to enroll 119 participants, of them, 79 in the NAC group and 40 in the placebo group.

### Randomisation and double-blind

Allocation will follow a computer-generated block randomisation list compiled by a biostatistician. The randomisation drug numbers will be sealed in opaque envelopes. All envelopes will be filed with the corresponding CRFs after use. Researchers and patients will not be informed about the specific randomisation. To ensure concealment of allocation, a study designer who is not involved in the trial implementation and data analysis is responsible for the randomisation list. Unblinding and data analysis will be started after all the participants finish the follow-up.

### Bias control

The inclusion and exclusion criteria of this study are clearly designed to reduce selection bias. Detailed data collection and strict quality management procedures will be formulated. All procedures are in accordance with the unified standard operating procedure (SOP), and trial-related tests will be conducted by experts at West China Hospital of Sichuan University to ensure the consistency of the results and reduce information bias. The final outcomes will be analysed by comprehensive statistical methods such as standardization, multivariate analysis, and propensity scoring to control for confounding factors.

### Dissemination plans

The results will be published in high-quality peerreviewed journals at the end of study.

## Discussion

NINCFB is being conducted to reveal the long-term efficacy of NAC in QoL and exacerbations of patients with NCFB and to explore the influence of NAC on airway bacterial colonization, chronic inflammation, oxidative stress and other manifestations.

Although NCFB is receiving increasing attention from patients and clinicians, there are a few relevant trials on its evaluation, management and treatment [1, 9]. According to investigations on Web of Science, nearly 520 articles of NCFB were published between 2012 and 2022, but less than 25 of them are randomised controlled clinical trials (RCTs) [1]. More high-quality evidence needs to be established for diagnosis and management of NCFB. This study is a multicentre, double-blind, randomised, placebo-controlled trial designed to provide new evidence for NCFB therapy and supervision.

Low QoL and repeated exacerbations cause distress in patients with NCFB and greatly increase their economic burden, and these would be the primary problems we should solve. Bacterial infection and airway bacterial colonization are considered the main causes of exacerbations of bronchiectasis not due to cystic fibrosis [38]. However, drug resistance and difficulty in eradicating biofilms with systemic antibiotics have led physicians to consider the possible role of nonantibiotic therapy [17].

Accordantly, the usage of expectorant might help to relive the continual expectoration and thus improve the life quality and reduce exacerbations in patients with NCFB, however, the relevant RCTs with comprehensive efficacy evaluation are limited (Table 4). Among the reported expectorants, the usage of inhaled mannitol and nebulised hypertonic saline showed no substantial improvement effects on the SGRQ score and exacerbations of patients with NCFB. By contrast, given the pharmacological action of NAC in antibiosis [17], researchers in Shandong Province of China have designed and conducted a relatively long-term (12 months) randomised controlled trial to demonstrate that NAC decreased the number of exacerbations in patients with NCFB [21]. However, they only included the patients with postinfection and idiopathic bronchiectasis, but not patients caused by immune deficiency and congenital defects, who also take NAC regularly to clear airway mucus in clinical practice; In addition, this study did not investigate the long-term performance of NAC in antibiosis, anti-inflammation and antioxidation; Furthermore, the therapeutic discrepancy of NAC among patients with different pathogenic (other than postinfection and idiopathic bronchiectasis), airway bacterial colonization, QoL scores, lung functions and exacerbation/hospitalization frequency were not assessed. As there is no expectorant proven to exert long-term efficacy against NCFB, a clinical trial to investigate the long-term therapeutic effects of NAC on the patients with NCFB is urgently needed. Consequently, to provide more comprehensive and stronger evidence for the benefits of NAC, our trial will not only evaluate the long-term efficacy of NAC on QoL and exacerbations of patients with NCFB, but also determine its effects on the pulmonary microbiology, inflammation, oxidative stress and identify the discrepancies of NAC efficacy related to disease heterogeneity.

**Table 4:** Previous RCTs reporting the effects of expectorant on NCFB patients

Study	Expecto-rants	Groups	Participants	Findings
Bilton et al. 2013 [39]	Mannitol	^a^E: inhaled mannitol 320 mg, twice a day for 12 weeks (*n* = 231) ^a^C: inhaled placebo 320 mg, twice a day for 12 weeks (*n* = 112)	1) 15–80 years of age 2) Clinically stable (for ≥2 weeks prior to study entry) and persistent cough 3) Chronic sputum (> 10 mL/d on the majority of days in the 3 months prior to enrollment) 4) Chronic chest congestion (chronic excessive accumulation of mucus) 5) FEV_1_ > 50% predicted 6) Having no active signs of asthma, malignancy, or TB, or uncontrolled hypertension, or smoking history.	There was no statistical difference between the groups in total SGRQ score. The changes in sputum weight over 12 weeks between mannitol and placebo group had significant difference, which appeared to be associated with increased antibiotic use in the placebo group.
Bilton et al. 2014 [40].	Mannitol	E: inhaled mannitol 400 mg, twice a day for 52 weeks (*n* = 233) C: inhaled mannitol 50 mg, twice a day for 52 weeks (*n* = 228)	1) 18–85 years of age; 2) Having at least two exacerbations in the previous 12 months; 3) Having a minimum SGRQ score of 30; 4) Producing at least 10 g of sputum a day; 5) FEV_1_ ≥ 40% and ≤ 85% predicted; 6) Having no mannitol-induced bronchospasm.	The decreasing in total SGRQ score over the treatment period in E group was more obvious than C group, while the changes in annual exacerbation rate, sputum weight, FEV_1_, and FVC between the two groups had no difference.
Kellett et al. 2011 [41].	Hypertonic saline	E: nebulised 7% hypertonic saline 4 mL, once daily for 12 weeks (*n* = 28) C: nebulised 0.9% sodium chloride 4 mL, once daily for 12 weeks (n = 28)	1) ≥ 18 years of age; 2) Having no bronchoconstrictor response to 7% hypertonic saline.	The changes in SGRQ score between the groups had no significant difference, while FEV_1_ and FVC during experimental phases had improved.
Nicolson et al. 2012 [42].	Hypertonic saline	E: nebulised 6% hypertonic saline 5 mL, once daily for 48 weeks (*n* = 20) C: nebulised 0.9% sodium chloride 5 mL, once daily for 48 weeks (n = 20)	1) ≥ 18 years of age; 2) Clinically stable; 3) Producing sputum daily 4) Reported ≥2 exacerbations requiring antibiotics per year for the previous 2 years. 5) FEV_1_ decreased by less than 15% after the hypertonic saline challenge and their FEV_1_ was more than 1 L at baseline.	The changes in SGRQ score, exacerbation rate, FEV_1_, and FVC between the two groups during 48 weeks had no difference.
Qi et al. 2019 [21]	NAC	E: NAC 600 mg, twice a day for 48 weeks (*n* = 81) C: as-needed therapy for 48 weeks (*n* = 80)	1) Diagnosis of idiopathic or post-infective bronchiectasis; 2) 18–80 years of age; 3) Clinically stable; 4) ≥ 2 exacerbations in the past year; 5) Having no cigarette smoking within 6 months; 6) FEV_1_ > 30% predicted 7) Having no active signs of asthma or malignancy.	The exacerbation frequency over the treatment period and 24-h sputum in E group was lower than C group. FEV_1_ and FVC between the two groups had no difference.

*TB* tuberculosis

^a^“E” means experimental group while “C” means control group

## Data Availability

The corresponding results will be published in high-quality journals after analysis. The raw datasets used and analysed during the current study will be available from the corresponding author on reasonable request.

## Abbreviations

NCFB: Non-cystic fibrosis bronchiectasis
NAC: N-acetylcysteine
QoL: Quality of life
COPD: Chronic obstructive pulmonary disease
ABPA: Allergic bronchopulmonary aspergillosis
NTM: Active nontuberculous mycobacterium
SGRQ: St George’s Respiratory Questionnaire
BSI: Bronchiectasis severity index
mMRC: Modified British Medical Research Council
HRCT: High-resolution computed tomography
FEV1: Forced expiratory volume in 1 s
CRF: Case report form
IL: Interleukin
TNF: Tumour necrosis factor
MMP: Matrix metalloproteinase
MPO: Myeloperoxidase
TAC: Total antioxidant capacity
SOD: Superoxide dismutase
MDA: Malondialdehyde
